# Surgically managed symptomatic intraspinal lumbar facet synovial cyst outcome of surgical treatment with resection and instrumented posterolateral fusion, a case series

**DOI:** 10.1186/s12893-022-01712-x

**Published:** 2022-07-15

**Authors:** Lyonel Beaulieu Lalanne, Roberto Larrondo Carmona, Juan I. Cirillo Totera, Facundo Alvarez Lemos, José Tomás Muñoz Wilson, Andre M. Beaulieu Montoya

**Affiliations:** 1 Orthopaedic Spine Surgeon, Head of Spine Center, Clínica Universidad de los Andes, Santiago, Chile; 2 Orthopaedic Spine Surgeon, Clínica Universidad de los Andes, Santiago, Chile; 3 Orthopaedic Spine Surgeon, Hospital del trabajador, Santiago, Chile; 4 Resident of Orthopedics and Traumatology, Clínica Universidad de los Andes, Santiago, Chile; 5 Universidad de los Andes, Santiago, Chile; 6 Las Condes, Los Trigales 7887, dep: 508, Santiago, RM Chile

**Keywords:** Synovial cysts (SC), Symptomatic synovial cysts (SSC), Conservative management (CM), Decompression and excision without fusion (DwoutF), Decompression and excision with fusion (DF)

## Abstract

**Background:**

There is controversy regarding the treatment of symptomatic synovial cysts, specifically, the need for a concomitant fusion when surgical resection of the synovial cysts is required. We present a retrospective review of a series of patients treated for symptomatic synovial cysts of the lumbar region during the last 20 years by a single surgeon, analyzing the current available literature.

**Methods:**

Retrospective review. The same surgical technique was applied to all patients. Demographic, clinical, surgical data and synovial cyst recurrence rate were recorded. Postoperative results reported by patients were documented according to the McNab score.

**Results:**

Sixty nine subjects, with mean follow-up of 7.4 years. 62% (43) were female, with a mean 57.8 years at the time of surgery. In 91.3% (63), the primary management was conservative for a minimum period of 3 months. All subjects underwent surgery due to the failure of conservative treatment. The segment most operated on was L4–L5 (63.77%). 91.3% (63) of the sample reported excellent and good and 6 subjects (8.6%) fair or poor results. There was no evidence of synovial cysts recurrence at the operated level.

**Conclusion:**

In symptomatic synovial cysts, it seems that conservative treatment is only effective in a limited number of patients and in the short term. Thus, the recommendation of a surgical indication should proceed as soon as the conservative management fails to result in significant symptom relief. Based on our results, we recommend, together with the resection of the cyst, the instrumentation of the segment to avoid its recurrence and the management of axial pain.

## Background

Juxtafacet cysts arise from the zygapophyseal capsule and may present as either intra or extraspinal structures. Vosschulte and Borger were the first to report nerve structure compression secondary to cysts in 1950. Such cysts are currently known as synovial cysts [1] (SC).

SC’s occur in the adult population with a higher prevalence among individuals older than 70, and according to Doyle and Merrilees [2], they are present in as much as 10% of patients with lumbar pain or radicular pain. However, their real prevalence worldwide remains uncertain and is probably higher than estimated in various studies.

This condition arises from a degenerative process of the joint, in which joint effusion together with facet joint arthrosis generate intraarticular fluid extravasation resulting in a facet capsule dilatation and protrusion towards the extraarticular space. In view of their etiology, Goffin describes such condition as “degenerative spinal joint cysts” that differentiate from ganglion cysts only histologically by the presence of a synovial lining [3].

Association with degenerative spondylolisthesis with some degree of instability is common and occurs most frequently at L4–L5 segment, the most mobile level of the spine, thus favoring instability of the segment and consequently degenerative changes that result in SC formation [4].

Regarding their clinical presentation, it is variable and depends on their location, size and relation to adjacent neural structures. Thus, they may present as an imaging finding in asymptomatic patients, mainly when they have an extracanal location, or they may cause symptomatic radicular clinical pictures similar to pulpous nucleus herniation or lumbar stenosis when they have an intracanal location. Moreover, cauda equina presentation due to SC has been reported in literature [5, 6].

When they present as symptomatic SC (SSC), they require treatment either conservative or surgical [7]. The latter is currently subject to debate, with the optimal approach still lacking consensus.

The purpose of the present work is to submit a retrospective review of a series of patients treated for lumbar region SSCs during the last 20 years by a single surgeon, and to analyze the current literature available.

## Materials and methods

Data from 69 patients with SSC who were operated on by the main author—L.B.L.- between November 1999 and September 2019, were retrospectively retrieved using the electronic record system in accordance with relevant guidelines and regulations (for example, Declaration of Helsinki), prior to obtaining approval from the ethics committee of our institution (Clinica Universidad de los Andes) and with the informed consent of the participants, in order to evaluate the results obtained.

Information gathered included demographic data, symptoms referred by the patient and findings of the preoperative physical examination, the treated segment, postoperative complications and cyst recurrence (defined as symptom recurrence together with imaging confirmation).

**Fig. 1:**
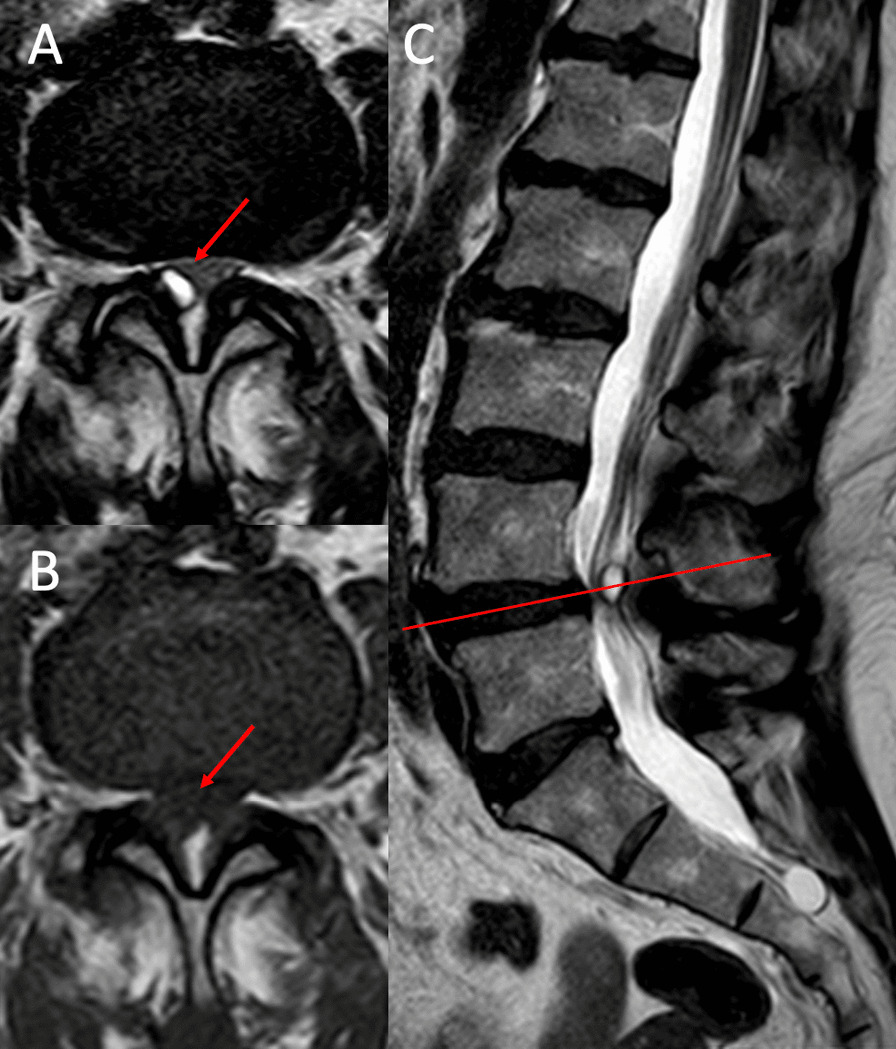
Synovial cyst at L4–L5 level in MRI. **A** Axial level cut in T2 sequence. **B** Axial section in T1 sequence. **C** Sagittal section in T2 sequence

All patients underwent a preoperative examination with standard flexion–extension X-ray (to assess segment instability defined as translation greater than 3 mm or 10º change in disc angulation [5]) and magnetic resonance imaging of the lumbar spine. Diagnosis was confirmed through visualization of an intracanal cystic mass adjacent to facet joints, hypointense in T1 and hyperintense in T2 (Fig. 1).

The same surgical technique was applied in every study individual, consisting of hemi-laminectomy and partial resection of the compromised facet with subsequent resection of the SC, enabling the release of the involved root and subsequent pedicle instrumentation of the operated segment with posterolateral arthrodesis, using an autograft derived from the decompression with ground tissue bank allograft and demineralized bone matrix (DBM), bilaterally in the same volume.

All patients were assessed postoperatively after 1 month, 3 months, 6 months, 1 year and once a year. The McNab Score (Table 1) was used to document surgical results of the operated individuals. The score was applied at the end of the follow-up period either at the outpatient clinic or by telephone. The score indicates 4 possible results: excellent, good, fair and poor.

**Table 1:** McNab Score, for postoperative functional outcome

Result	Criteria
McNab score	
Excellent	Neither pain nor restriction of mobility
Good	Lumbar or lower limb pain of sufficient severity to interfere with the patient’s ability to do normal work
Fair	Improved functional capacity but handicapped by intermittent pain of sufficient severity to interfere with work or leisure activities
Poor	No improvement or insufficient improvement to enable increase in activities carried out prior to surgery

## Results

Of the 69 patients who underwent surgery for SSC and answered the postoperative evaluation questionnaire, they had at least one year of follow-up (range: 1–20 years, mean 7.4 years).

Forty-three patients (62%) were female, and the age ranged between 36 and 79 with a mean age of 57.8 at the moment of surgical intervention.

All the patients presented with radicular pain as a result of compression of neural structures. Additional axial lumbar pain was referred by 71% (49 patients).

**Fig. 2:**
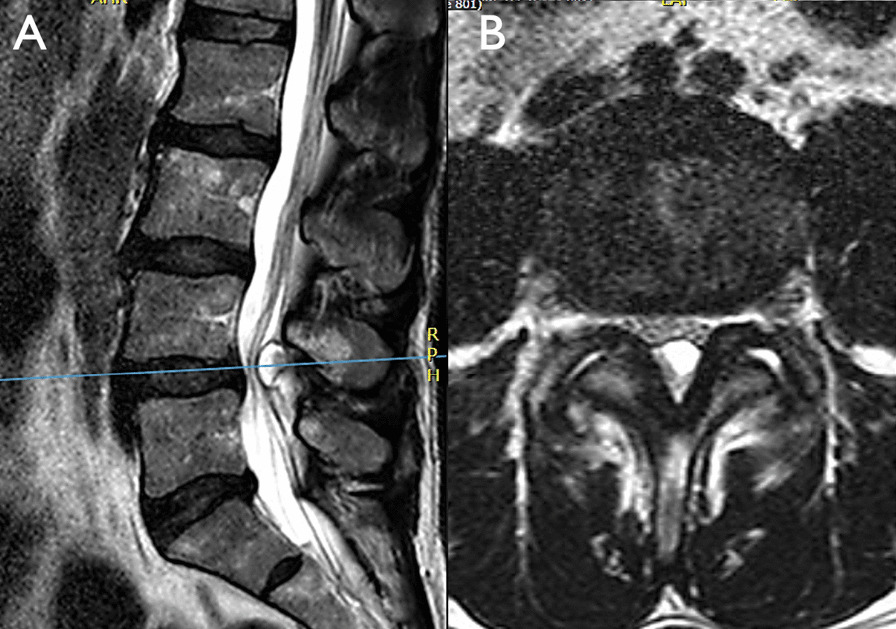
Synovial cyst at L4–L5 level in MR. In sagittal section (**A**) and axial section (**b**), both in T2 sequence, of one of the patients with a cauda equina syndrome

Surgery was selected as the initial therapy in six patients (8.7%), two of which (2.9%) underwent an emergency operation due to a presentation consistent with a Cauda Equina Syndrome (Fig. 2). The remaining four patients (5.8%) had an indication of early surgical decompression due to a radicular neurological deficit either equal or lower than M3 or progressive.

**Fig. 3:**
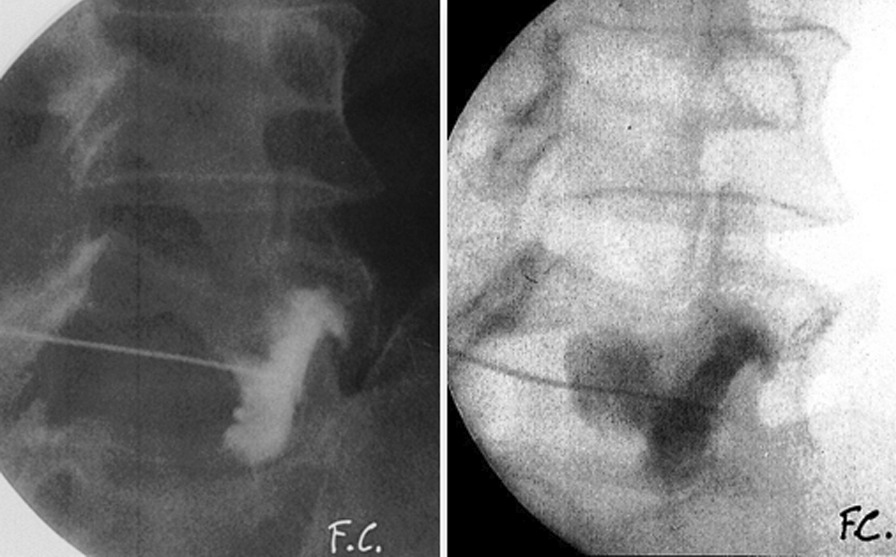
Zygapophyseal infiltration under fluoroscopic guidance with contrast medium, confirming CS rupture by observing contrast extravasation

In the remaining 63 patients (91.3%) a primary conservative management (CM) approach, driven by rest, analgesia and physical therapy was implemented for a minimal duration of 3 months. Such patients also underwent a zygapophyseal infiltration under fluoroscopic guidance with contrast medium, aiming at percutaneous rupture of the SC that was confirmed in 49 individuals (77.8%) through the visualization of contrast medium extravasation and loss of pressure of the plunger (Fig. 3). A second infiltration was carried out on 14 patients (22.2%) who had either persistent or recurrent pain within the first 8 h after the first procedure. Ultimately all individuals underwent surgical intervention due to a failure of medical therapy or as a result or symptom recurrence.

The most frequently operated segment was L4-L5, in a total of 44 patients (63.77%), followed by segment L3–L4 in 16 patients (23.19%). Only 8 individuals (11.59%) were operated in segment L5–S1.

**Fig. 4:**
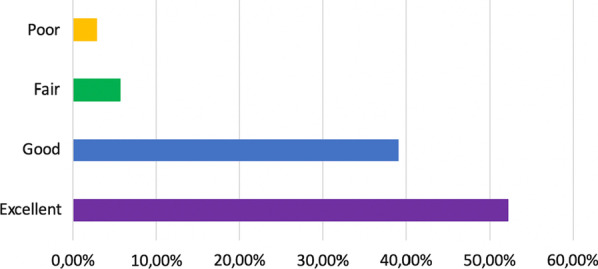
Postoperative result according to McNab score

As for the McNab criteria, 91.3%, 63 patients, referred excellent and good outcomes (52.2% and 39.1%, respectively), at the end of the follow up period. Of the whole sample, 97.1% related a complete relief of their previous radicular pain immediately after surgery. Of those patients who also suffered axial lumbar pain, 87.5% presented relief of the latter within the first postoperative 8 weeks. Six individuals (8.6%) of patients related fair or poor outcomes (Fig. 4).

Two incidental durotomies occurred during surgery and were documented for the first two patients of the series. These were repaired during the same surgical opportunity and had a favorable outcome, without sequel after strict rest for 48 h.

Seven postoperative complications (10.1%) were observed. Three were radiculitis that underwent CM (rest and combined therapy with non-steroidal-anti-inflammatory drugs, pregabalin and oral steroids), two of which progressed with full symptom relief within three weeks. The third patient required a selective radicular block in two opportunities to finally achieve full recovery of the condition at the 4th postoperative month. The remainder of complications were seromas of the operative wound, that occurred in four patients.

Only one patient required a second-look surgery due to a transitional syndrome of the adjacent segment with development of a new SSC with onset two years after the initial surgery. Neither other second-look surgeries nor SC recurrence at the operated level were observed in this case series.

## Discussion

SC are a common cause of lumbar pain among adults, presenting more frequently between the sixth and seventh decade of life, with a slight predominance among females [8]. Such characteristics coincide with those observed in our case series.

The current gold standard for imaging diagnosis of SC is magnetic resonance imaging [9], with a maximum reported sensitivity of 90% [10]. SSC presents as a compressive well-outlined, intracanal, extradural lesion, generally located adjacent to a facet joint, with higher intensity than cerebrospinal fluid (CSF) in T2 and low intensity in T1 [11]. However, signal intensity may vary according to the presence of protein and/or blood content within the cyst.

Of our sample, 63.77% of patients underwent instrumentation at the segment L4-L5, in accordance with the pathogenesis of SCs, since such level is the most involved [4] as a result of having the greatest mobility of the lumbar spine and because of the instability generated there as part of the vertebral spine degenerative process. The latter also explains why this pathology is much less common at the dorsal spine, which is rigid and motionless [12].

Presently, there is still no consensus regarding the optimal treatment of this condition, which is a constant subject of debate. Although cases of SSC with spontaneous remission have been reported, some kind of therapy is generally needed for symptom resolution.

The first line of treatment is CM, which is based on a combination of rest, analgesics and physical therapy, and it is indicated in patients without progressive or significant neurological deficit. However, results reported in the literature are not fully satisfactory [3, 4]. Metellus et al. [11], conducted a retrospective study where they analyzed functional outcomes of 77 patients treated conservatively and observed a failure rate of 60% at six months. Likewise, Parlier-Cuau et al. [13], in their retrospective series of 30 cases, reported only 33% of excellent or good outcomes when using the CM and they had to choose surgical intervention in 47% of their sample. In 2003, Shah and Lutz [4] carried out a literature review and identified 139 patients treated with a CM, in which 47% had to undergo surgical treatment as a result of an unsuccessful CM.

Another option within the CM is facet infiltration with steroids during the attempt to aspirate or rupture the cyst through a percutaneous approach. However, results do not seem to be better than those seen in other CM modalities. Allen et al. [14], in 2009, retrospectively analyzed 32 patients that underwent percutaneous rupture of the SC. A symptom relief was observed in 72% of patients at one year of follow-up. However, 37.5% presented cyst recurrence at 3 months and 55% required surgery for cyst resection. On the same year, Martha et al. [15] evaluated 101 patients who had undergone the same procedure, with confirmed cyst rupture in 81% of patients. Likewise, 54% of patients had to undergo surgical intervention after an average of 8 months. Other earlier studies such as the one by Parlier-Cuau et al. [13], report similar outcomes; and in case series in which steroid infiltration was selected, success rates were not higher than 57% [16].

In our case series, 63 patients underwent steroid infiltration and cyst rupture, which was confirmed in 49 patients (77.8%). Although the main purpose of our study is not to assess the efficacy of the CM, as in the previously mentioned studies, individuals did not show long-term symptom relief, and had to be operated. Therefore, surgical intervention is recommended in patients in which CM does not evidence a significant improvement [8].

Surgical technique and fusion together with instrumentation may vary depending on the location and the relation of the cyst to neural structures and the presence of concomitant local pathologies. Nevertheless, they remain a motive of debate.

Certain authors propose that the surgery of choice is a hemilaminectomy and partial facetectomy and cyst excision, without fusion (decompression and excision without fusion—DwoutF), the latter being the less invasive approach. Eventually, the potential risk entailed by such technic is the generation or increase of the instability of the compromised segment, that might theoretically increase recurrence of the condition or generate a chronic lumbar pain.

SC recurrence rates (development of a new SC at the same level, after surgical removal) have been reported to range from 15 to 25% among patients undergoing DwoutF procedures [17, 18], while case series in which decompression and excision with fusion (DF) was carried out reported rates close to 0% [19, 20], data in accordance with our case series.

The systematic review of the literature by Bydlon et al. [21], identified 966 patients of which 84% of these were treated with surgical DwoutF, reporting only a cystic recurrence rate of less than 2% and with postoperative back pain in 21.9% of the subjects, after a minimum follow-up of two years.

In 2000, de Lyons et al. [9] published a retrospective study in which 194 patients with SC were evaluated. Their results did not evidence a correlation between the degree of laminectomy and/or facetectomy and the development of postoperative symptomatic spondylolisthesis. Likewise, Trummer et al. [16] and Sabo et al. [22] failed to find significant differences between the different surgical techniques and the final outcome. They concluded that the requirement of DF will depend on the previous segment degree of instability and promoted the use of flexion/extension X-rays to assist in evidencing the instability.

It would appear that the association of isolated cyst resection with an increased risk of segmental instability is not entirely clear. Instrumented fusion must be targeted to the instability of the segment to be operated. Such instability may be identified in dynamic (flexion/extension) X-rays as a displacement greater than 3 mm or more than 10º angulations between adjacent vertebral bodies [22, 23]. Blumenthal et al. [24], also recommend an instrumented arthrodesis in patients with a facet angle greater than 50º, disc height higher than 6.5 mm and a displacement greater than 1.25 mm between vertebrae.

Indirect signs of instability evidenced both in MR as well as in CT scan, such as intradiscal or intraarticular vacuum phenomenon, ligament flavum hypertrophy or presence of more than 1.5 mm effusion [25] within the involved facet joints in conjunction with the presence of axial pain, might also strengthen the indication for instrumentation of the segment to be operated.

The requirement for an DF should be assessed individually in each case as it adds risks compared to DwoutF (longer hospital stay, higher risk of incidental durotomy, higher blood loss and higher rates of perioperative infection) [21, 22].

Finally, advances in minimally invasive surgery techniques have allowed the resection of the SC with less damage to the posterior stabilizing structures [21], showing good results [26, 27], however there is still a lack of bibliography with a higher level of evidence to be able to determine if these procedures represent a significant advantage as well as to be able to determine which patients really need DwoutF.

The present study has an adequate number of patients, considering the low prevalence of this pathology, who were operated on by the same surgeon and with the same surgical technique, resulting in a homogeneous sample and a non-negligible follow-up period. The main limitation is the retrospective design, and the absence of a control group to contrast outcomes. The latter restricts the possibility of other analyses with our results. Also, in some patients the follow up completion was, by telephone, thus adding a memory bias to the study. prospective randomized studies with a control group are needed to assess the real association between good results and lower recurrence rate among patients undergoing instrumented arthrodesis.

## Conclusion

SCs represent a frequent pathology in the adult population, closely related to spondylolisthesis, with the L4–L5 segment being the most frequently affected. MRI is usually sufficient for diagnosis.

When such a condition is symptomatic, CM appears to be effective only in a limited number of patients and for a short period of time. Even with joint infiltrations and percutaneous cyst rupture, the effectiveness rates seem to remain unchanged and therefore the recommendation for a surgical indication should proceed as soon as CM does not produce significant relief of symptoms.

Current literature fails to demonstrate differences in results for DwoutF or DF. However, according to our results, we recommend the evaluation of each patient, with dynamic radiographs and magnetic resonance imaging to assess the instability of the segment and in such cases, perform FD to prevent recurrence in the operated segment and manage axial pain.

## Data Availability

The datasets used and/or analyzed during the current study are available from the corresponding author.

## Abbreviations

SC: Synovial Cysts
SSC: Symptomatic Synovial Cysts
CM: Conservative management
DwoutF: Decompression and excision without fusion
DF: Decompression and excision with fusion
